# A database of global storm surge reconstructions

**DOI:** 10.1038/s41597-021-00906-x

**Published:** 2021-05-04

**Authors:** Michael Getachew Tadesse, Thomas Wahl

**Affiliations:** Civil, Environmental, and Construction Engineering & National Center for Integrated Coastal Research, University of Central Florida, Orlando, USA

**Keywords:** Natural hazards, Physical oceanography

## Abstract

Storm surges are among the deadliest coastal hazards and understanding how they have been affected by climate change and variability in the past is crucial to prepare for the future. However, tide gauge records are often too short to assess trends and perform robust statistical analyses. Here we use a data-driven modeling framework to simulate daily maximum surge values at 882 tide gauge locations across the globe. We use five different atmospheric reanalysis products for the storm surge reconstruction, the longest one going as far back as 1836. The data that we generate can be used, for example, for long-term trend analyses of the storm surge climate and identification of regions where changes in the intensity and/or frequency of storms surges have occurred in the past. It also provides a better basis for robust extreme value analysis, especially for tide gauges where observational records are short. The data are made available for public use through an interactive web-map as well as a public data repository.

## Abstract (summary)

**Table:** 

Measurement(s)	storm surge
Technology Type(s)	Data Driven Analysis • computational modeling technique
Factor Type(s)	oceanographic and meteorologic predictors
Sample Characteristic - Environment	coastal sea water
Sample Characteristic - Location	global coastal zone

Machine-accessible metadata file describing the reported data: 10.6084/m9.figshare.13296035

## Background & Summary

Understanding the stochastic nature of extreme storm surges and how they are affected by climate change and variability is important for efficient design of coastal defense structures and planning of future coastal adaptation^1^. A major hurdle in assessing long-term trends in the storm surge climate and performing robust statistical analyses is the lack of sufficiently long data records^2,3^.

Taking extreme value analysis as an example, the rule of thumb is that extrapolation of extreme water level events should be limited to return periods not longer than four times the available record length^4^. This means in order to derive an estimate of the 100-year storm surge level, at least 25 years of data are needed (to derive a 500-year event, 125 years of data would be needed). However, tide gauge records, which are the main data source for storm surge information, often cover much shorter time periods. From the global dataset of extreme sea level observations we used, 45% of the 882 tide gauges included in this study have less than 25 years of data. Moreover, analyzing long-term trends and multi-decadal variability in the storm surge climate is also hampered by short record lengths. To assess the long-term trend in global mean sea level it has been estimated, for example, that a minimum of 60 years of data are required^5^, which is the case for only 12% of the tide gauges analyzed here; given the larger variability, even longer records are needed to reliably explore long-term changes in the storm surge climate.

Hydrodynamic storm surge models have recently been developed and applied to simulate storm surges globally for the past and future, but the computational burden of running these models is high, limiting the possibility to create very long hindcasts (currently simulations only go back to 1979)^6,7^. In light of these challenges and to overcome this limitation of large-scale hydrodynamic models, data-driven statistical approaches have been used to develop long-term storm surge reconstructions (as far back as 1866) at the regional^8^ and global scale^9^. The statistical models are trained with predictors from climate reanalysis and observed water levels at tide gauge locations. Both paradigms, data-driven methods as well as hydrodynamic models, have their own advantages and disadvantages (see Tadesse *et al*.^10^ for more information).

We present here the Global Storm Surge Reconstruction (GSSR) database making use of already developed data-driven models^10^ and multiple satellite-era as well as longer-term atmospheric reanalysis products, going as far back as 1836. The long-term surge reconstruction we present here can be used for more robust extreme value analysis, especially in locations where observational records are short, as well as to better understand the trends and longer-term variations in the storm surge climate (e.g., intensity and frequency of storm surge events) from the mid-nineteenth century until present (please see Usage Notes).

**Fig. 1:**
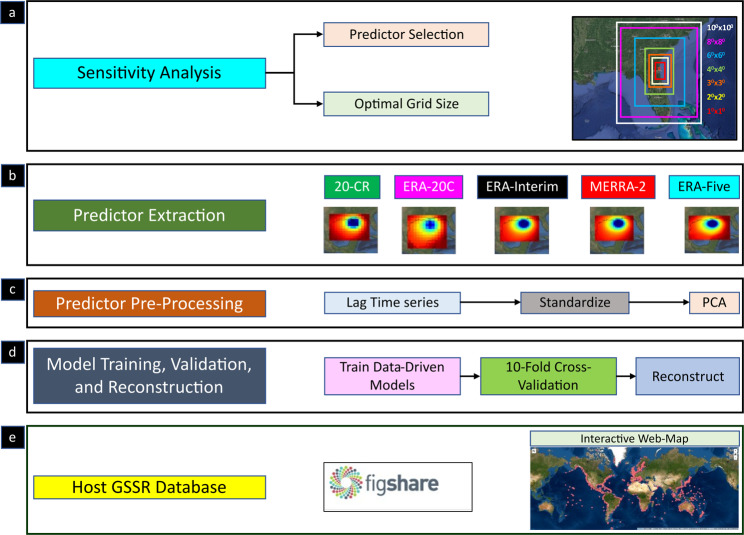
Schematic diagram of the overall workflow implemented to produce the GSSR database. The different steps include sensitivity analysis (**a**), predictor extraction (**b**), predictor pre-processing (**c**), model training, validation and reconstruction (**d**) and hosting the GSSR database (**e**).

The data-driven models employed for the surge reconstruction were developed by Tadesse *et al*.^10^ (hereinafter referred to as T20) by using multiple linear regression and Random Forest at each tide gauge location to derive daily maximum surge values. The overall workflow for the database development is outlined in Fig. 1 and the individual steps are discussed in more detail below.

## Methods

### Data sources

The training component of our storm surge reconstruction methodology employs two major input datasets, namely predictors and predictands. Predictors in T20 are atmospheric and oceanographic variables, including zonal (u10) and meridional (v10) wind speeds, mean sea-level pressure (SLP), sea-surface temperature (SST) and precipitation. We utilize five medium to long-term climate reanalysis products (Table 1) in order to extract relevant predictors (see Sensitivity Analysis for details). The necessary pre-processing steps are applied on predictors obtained from each reanalysis product (see Predictor Pre-processing for details).

**Table 1:** Metadata for the climate reanalysis datasets used for surge reconstruction.

Name	Source	Spatial resolution	Temporal resolution	Coverage
ERA-20C	ECMWF	1.125° × 1.125°	3 hourly	1900–2010
20-CR	NOAA-CIRES-DOE	1.0° × 1.0°	3 hourly	1836–2015
ERA-Interim	ECMWF	0.75° × 0.75°	6 hourly	1979–2019
MERRA-2	NASA	0.625° × 0.5°	1 hourly	1980–2019
ERA5	ECMWF	0.25° × 0.25°	1 hourly	1979–2019

The atmospheric reanalyses we use to produce GSSR (Table 1) can be categorized into two categories. The first category comprises satellite-era reanalyses including ERA-Interim^11^ and ERA5^12^ from the European Centre for Medium-Range Weather Forecasts (ECMWF) and the Modern Era Retrospective analysis for Research and Applications (MERRA-2)^13^ from the National Aeronautics and Space Administration (NASA). The second category comprises centennial reanalyses including the 20^th^ Century Reanalysis (20-CRV3)^14^ – a joint product from the National Oceanic and Atmospheric Administration (NOAA), Cooperative Institute of Research in Environmental Sciences (CIRES), and U.S. Department of Energy (DOE) – as well as ERA-20C^15^ from ECMWF. The centennial reanalyses have a coarser spatial (see Fig. 1b; the sea-level pressure from Hurricane Katrina) and in most cases lower temporal resolution than the satellite-era reanalyses, but they allow the reconstruction of longer storm surge time series. The web links to the data repositories from which we downloaded the reanalysis datasets are provided in a README file pertaining to the resulting surge reconstructions (see Table 2 for the DOIs to access the different surge reconstructions).

**Table 2:** Information on the repository where each reconstruction is stored.

Data	DOI
20-CR Surge Reconstruction^23^	10.6084/m9.figshare.12971075
ERA-20C Surge Reconstruction^23^	10.6084/m9.figshare.12971054
ERA-Interim Surge Reconstruction^23^	10.6084/m9.figshare.12971090
MERRA-2 Surge Reconstruction^23^	10.6084/m9.figshare.12971009
ERA5 Surge Reconstruction^23^	10.6084/m9.figshare.12970931
All scripts used for GSSR^23^	10.6084/m9.figshare.12978191
Common Period Validation^23^	10.6084/m9.figshare.13416293
Online Interactive Web-Map	http://gssr.info

The predictand are daily maximum storm surge values at 882 tide gauges. Observed sea level data for individual tide gauges were obtained from the Global Extreme Sea Level Analysis (GESLA-2) database^16^. We remove annual mean sea-level from the hourly sea-level data. Daily maximum surge values (i.e., the predictand) are extracted by applying a harmonic tidal analysis to detrended hourly sea level, and deriving the maximum daily values of the residuals after the tidal signal was removed.

### Sensitivity analysis

The methodology used to develop the GSSR database is the same as outlined in T20. However, we carry out an additional two-phased sensitivity analysis (Fig. 1a) with the goal of simplifying the modelling approach and making it more efficient when reconstructing surge time series over long time scales with predictor data sets from multiple reanalysis products with varying spatial and temporal resolution.

**Fig. 2:**
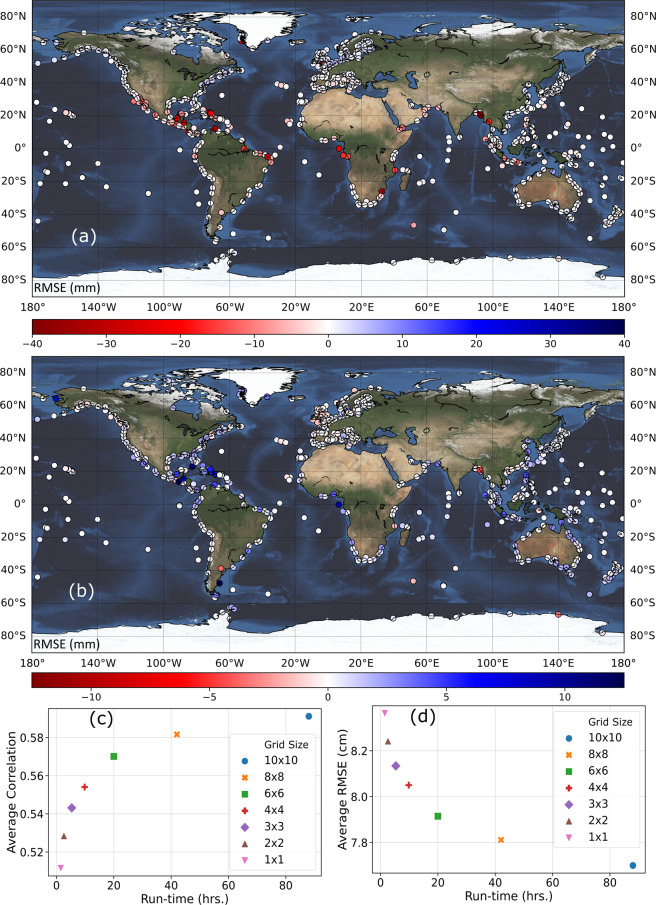
Sensitivity analysis to explore model simplifications. Changes in model accuracy, expressed as RMSE (mm), when adding precipitation (**a**) and SST (**b**) to the baseline model. Negative RMSE values (shown in red) indicate lower model accuracy when adding either precipitation or SST and positive RMSE values (shown in blue) indicate higher model accuracy when adding either precipitation or SST. Changes in model accuracy when using varying box sizes to derive predictor data expressed as correlation (**c**) and RMSE (**d**).

First, we explore a possible simplification by reducing the number of predictors used in T20 (10 m u10 and v10, SLP, precipitation, and SST). Conventionally, SLP and wind speed are the two main predictors used to model storm surges with statistical^8,9,17^ or numerical^18–20^ approaches. Hence, we explore whether dropping some of the predictors used in T20 has a significant impact on the results. We first establish a baseline scenario that only uses SLP, u10, and v10 as predictors (the training and validation procedure is the same as in T20). Then, we add precipitation as an additional predictor and compare the validation results against the baseline scenario (Fig. 2a). We repeat the same with SST (Fig. 2b). All predictors for this experiment come from the ERA-Interim reanalysis. At more than 92% of the tide gauges adding precipitation as a predictor changes the model accuracy, expressed as Root Mean Square Error (RMSE), by less than 5 mm; for SST, the same is true for 99% of the tide gauges. However, adding precipitation lowers RMSE more substantially for some tide gauges (e.g., New York – The Battery has a 7.3 mm reduction in RMSE, which is the highest reduction we find); such larger changes are very localized and could be the result of freshwater flows affecting the recorded surges at the tide gauges, which is not accounted for in our analysis when excluding precipitation. Similarly, there are tide gauges (mostly in the tropics) where larger RMSE reductions are found when including SST (e.g., at Puerto Deseado, Argentina, we find a 14 mm reduction). Nevertheless, the changes in model accuracy are negligible in the majority of locations, but removing SST and precipitation predictors leads to significant improvements in the efficiency of the model; hence, we chose the baseline model-setup to generate the GSSR database.

In the second phase of the sensitivity analysis, we identify the optimal area around tide gauges from where predictor information is considered to train/validate the data-driven models; T20 used a 10° × 10° box around every tide gauge. This approach, while feasible with the ERA-Interim reanalysis used in T20, leads to increased computational cost for MERRA-2 and especially ERA5 which have much higher spatial resolution (and in the case of ERA5 also higher temporal resolution). This significantly increases the amount of data that is considered to define the predictor time series. We explore here the optimal size of the area around a tide gauge that reconciles a tradeoff between model accuracy and computation time. To this end, we test box sizes of 1° × 1°, 2° × 2°, 3° × 3°, 4° × 4°, 6° × 6°, and 8° × 8° and train/validate the data-driven models and compare model accuracies with the baseline case that uses a 10° × 10° box. The results (Fig. 2c,d) indicate that an area of 6° × 6° is the optimal choice (i.e., shortest distance to upper left and lower left corners) considering model accuracy (measured in RMSE and Pearson’s Correlation) and computational time (measured in hours). The latter includes the time for extracting the predictors and training and validating the model. Results are shown for ERA-Interim; differences in computation time for different box sizes are much larger for MERRA-2 and ERA5. Hence, we use a 6° × 6° box around each tide gauge when developing the GSSR database.

### Predictor extraction

Based on the findings from the sensitivity analysis, predictors (u10, v10, and SLP) are extracted from within a 6° × 6° box around each tide gauge from the netcdf files of the atmospheric reanalyses and stored in a comma-separated value (.csv) file. The satellite-era reanalyses have a higher spatial resolution and hence a much larger number of grid points. For instance, ERA5 contains around 576 grid points in a 6° × 6° box, whereas 20-CR contains only 36 grid points (see also Fig. 1b).

### Predictor pre-processing

Following the extraction of the three predictors, we concatenate all extracted files per predictor corresponding to each year. For instance, for ERA-Interim and ERA5 with 40 years of data we concatenate 40 years of predictor data to form one long time series for each predictor and tide gauge (see Table 1 for time periods covered by the different reanalyses). Next, the three predictor time series (u10, v10, and SLP) are combined into one matrix. Following T20 we lag this matrix of multiple predictors as far back as 30 hours in order to include the delay effect of predictors on daily maximum surge. After the time-lagging of predictors, we standardize the predictor matrix to account for the different units used by each predictor. At this stage, the predictor matrix is very large and it becomes a daunting task to apply further matrix operations, especially for the satellite-era reanalyses with high spatial and temporal resolution. For instance, the predictor matrix for ERA5 at this stage includes more than 24,000 features, whereas for 20-CR it includes approximately 2,700 features. In order to reduce dimensionality, Principal Component Analysis (PCA) is applied to extract only the principal components that explain 95% of the variance within the predictor matrix. This reduces the feature size of the predictor matrix to approximately 250, making further matrix operations more convenient. This step concludes the pre-processing stage (see Fig. 1c) and the predictor matrix can then be used for training and validation of the data-driven models.

### Model training, validation, and reconstruction

We use the pre-processed predictor matrix to train and validate the data-driven models following T20 (see Fig. 1d), using both multiple linear regression and Random Forest to link predictor and predictand data. Note, that only a portion of the predictor matrix is used for training/validation as the predictand (observed daily maximum surge) almost always has shorter coverage than the predictor data from the reanalyses. The 10-Fold cross-validation technique is used for validation. For each reanalysis dataset, we provide the validation of the data-driven models at each tide gauge location in terms of Pearson’s correlation coefficient as well as the RMSE:

$$\begin{array}{ccc}Pearson\mbox{'}s\;Correlation\,({r}_{xy}) & = & \frac{{\sum }_{i=1}^{n}({x}_{i}-\mathop{x}\limits^{-})({y}_{i}-\mathop{y}\limits^{-})}{\sqrt{{\sum }_{i=1}^{n}{({x}_{i}-\mathop{x}\limits^{-})}^{2}}\sqrt{{\sum }_{i=1}^{n}{({y}_{i}-\mathop{y}\limits^{-})}^{2}}}\\ {\rm{RMSE}} & = & \sqrt{\frac{{\sum }_{i=1}^{n}{({y}_{i}-{x}_{i})}^{2}}{n}}\end{array}$$

where *x* and *y* represent the values of the observed and modeled surges and $\mathop{x}\limits^{-}$ and $\mathop{y}\limits^{-}$, the mean of observed and modeled surges.

For the Technical Validation (see below) we also use the Relative RMSE (RRMSE) as an additional metric, following T20. RRMSE normalizes RMSE by the maximum surge variability observed at a specific tide gauge (difference between the maximum and minimum surge). This validation information can be found in the metadata section of the repository where the surge reconstructions are stored (refer Table 2). Once the training and validation stage is complete, the full predictor matrix is used to develop the surge reconstruction over the entire period where predictor information is available from the reanalysis datasets. As a measure of uncertainty, we provide the 95% prediction intervals for all simulated daily surge values.

## Data Records

The DOIs of the repositories for the surge reconstructions (derived with different reanalysis products) are shown in Table 2; included in these repositories are README files, folders that contain the surge reconstructions for all tide gauges, and metadata folders with model validation results for all tide gauges (both for daily maximum surges and extreme surges above the 95^th^ percentile). Daily max surge time series (along with 95% prediction intervals) are stored, for individual tide gauges, in comma-separated value (.csv) format.

## Technical Validation

We validate the surge reconstructions for the different reanalysis products and individual tide gauges using a 10-fold cross-validation. As the five reanalysis products have different start and end times, the validation periods (or fold sizes) for which the performance metrics are derived are also different (surge reconstructions derived with the longer-term reanalyses have longer validation periods). The validation results are provided in the metadata section of the corresponding surge reconstruction (refer Table 2 for the respective DOIs).

In addition to the separate validation for the different surge reconstructions, we also assess the variability in model accuracy among the five surge reconstructions for a specific tide gauge. This allows to investigate if there are any spatial patterns where certain reanalysis products lead to better results than others. To allow for a more direct comparison across all surge reconstructions, we select a common period (1980–2010) which is covered by all five reanalysis products. We validate the reconstructions during this period in terms of Pearson’s correlation, RMSE, and RRMSE, and show the variability (measured as standard deviation) of model accuracy (refer Table 2).

**Fig. 3:**
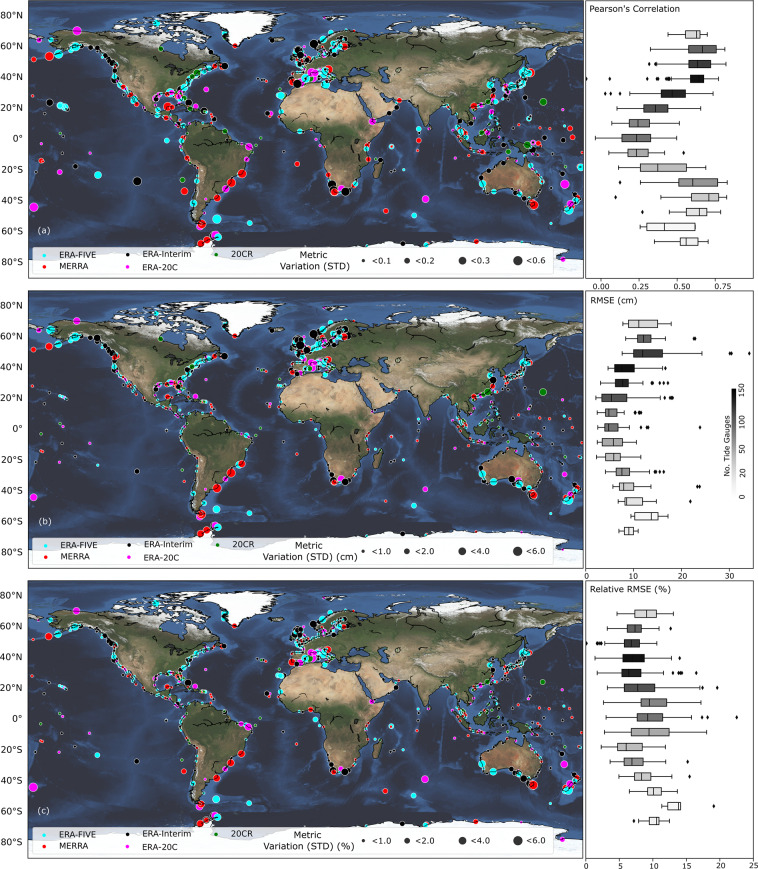
Model validation results. Validation of five surge reconstructions during 1980–2010 in terms of Pearson’s correlation (**a**), RMSE (**b**), and RRMSE (**c**). Color-coding indicates which reanalysis led to the best surge reconstruction and circle sizes indicate the magnitude of the standard deviation of the metrics across all reconstructions. Right panes show the variability of the metrics for the best surge reconstruction over 10 degree latitude bands. Tide gauges at latitudes greater than 65° N and lower than 65° S are included in the first and last boxplots respectively for each plot in the right panel, due to the small amount of tide gauges in these regions.

Figure 3, for instance, illustrates on the left panes the variability in the different performance metrics across the five surge reconstructions and depicts the respective reanalysis product that leads to the highest model accuracy. On the right panes, we show the spatial distribution (summarized in 10-degree bands) of the validation results for the surge reconstructions that lead to the highest model accuracy. This is in agreement with validation results reported in T20, including overall higher model accuracy in higher latitudes and lower accuracy in the tropics. We find an average RMSE of 9.9 cm (std = 4.1) for extratropical regions and 6.3 cm (std = 3.1) for tropical regions. Average correlation is 0.6 (std = 0.13) for extratropical regions and 0.33 (std = 0.16) for tropical regions. Moreover, at the vast majority of tide gauges the surge reconstructions derived with ERA-Interim or ERA5 lead to the best results (for 33% and 30% of tide gauges respectively). Notable exceptions are the east coast of South America, east coast of Australia, and parts of southeast Asia, where MERRA-2 leads to the best results at many locations. Overall, MERRA-2 leads to the best results at 18% of the tide gauges. At the remainder of locations ERA-20C (12%) and 20-CR (7%) lead to the best results.

**Fig. 4:**
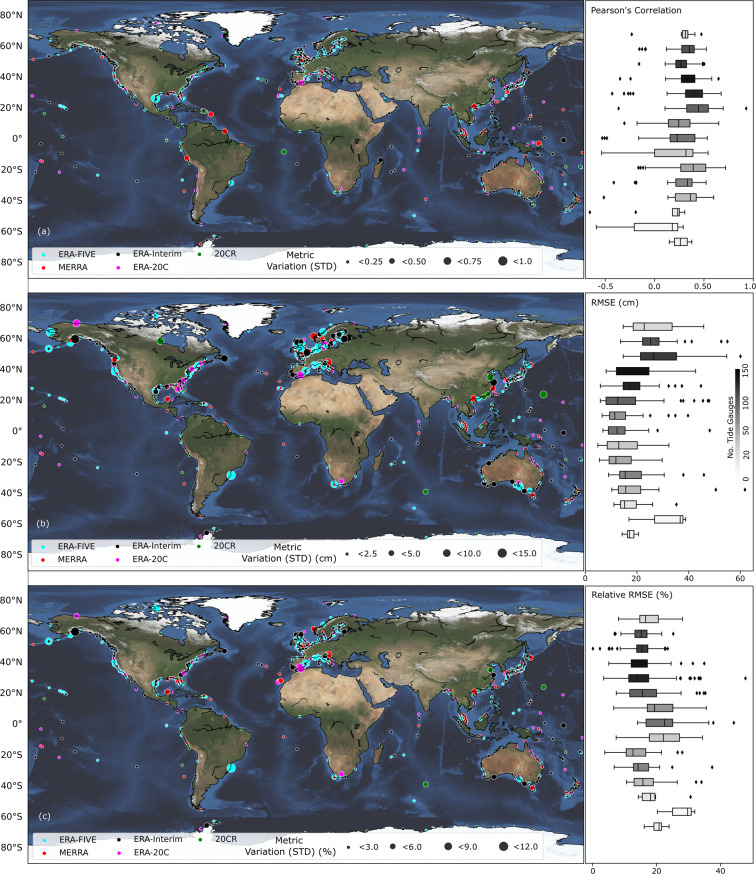
Model validation results for extreme events. Validation of five surge reconstructions during 1980–2010 in terms of Pearson’s correlation (**a**), RMSE (**b**), and RRMSE (**c**) for extreme surge events (above the 95% percentile). Color coding indicates which reanalysis led to the best surge reconstruction and circle sizes indicate the magnitude of the standard deviation of the metrics across all reconstructions. Right panes show the variability of the metrics for the best surge reconstruction over 10 degree latitude bands. Tide gauges at latitudes greater than 65° N and lower than 65° S are included in the first and last boxplots respectively for each plot in the right panel, due to the small amount of tide gauges in these regions.

We also compare the surge reconstructions with observations from the perspective of extremes. For each tide gauge, observed surges above the 95^th^ percentile threshold are compared with the simulated values (Fig. 4). When considering the 95^th^ percentile threshold, some tide gauges with short records have only very few data points leading to insignificant correlation coefficients; these are not shown in Fig. 4 and as a result the number of tide gauges in Fig. 4 is slightly lower than that of Fig. 3. We find an average RMSE of 22 cm (std = 9.9) for extratropical regions and 15 cm (std = 8.5) for tropical regions. Average correlation is 0.32 (std = 0.16) for extratropical regions and 0.31 (std = 0.25) in tropical regions. For 65% of tide gauges the same reanalysis that gives the best validation results for daily maximum surges also gives the best validation results for the extremes, whereas in 35% of the cases a different reanalysis leads to better results when only focusing on extremes (therefore, as outlined above, we provide validation results for both in the metadata).

When comparing the validation results across surge reconstructions at individual tide gauges, higher variability is found in higher latitudes in both hemispheres (Figs. 3 and 4, left panes), compared to tropical regions. This means that in high latitudes some reanalysis products lead to much better results than others, while in the tropics all reanalyses lead to similar results. This can be corroborated by the findings in T20 that there is overall more variance in wind speed and SLP in higher latitudes and this is reflected better in some reanalyses, leading to higher variability of model accuracy across reanalyses at individual tide gauge locations. In tropical regions, where predictor variability is smaller and model performance overall poorer, the differences across reanalyses are less pronounced.

**Fig. 5:**
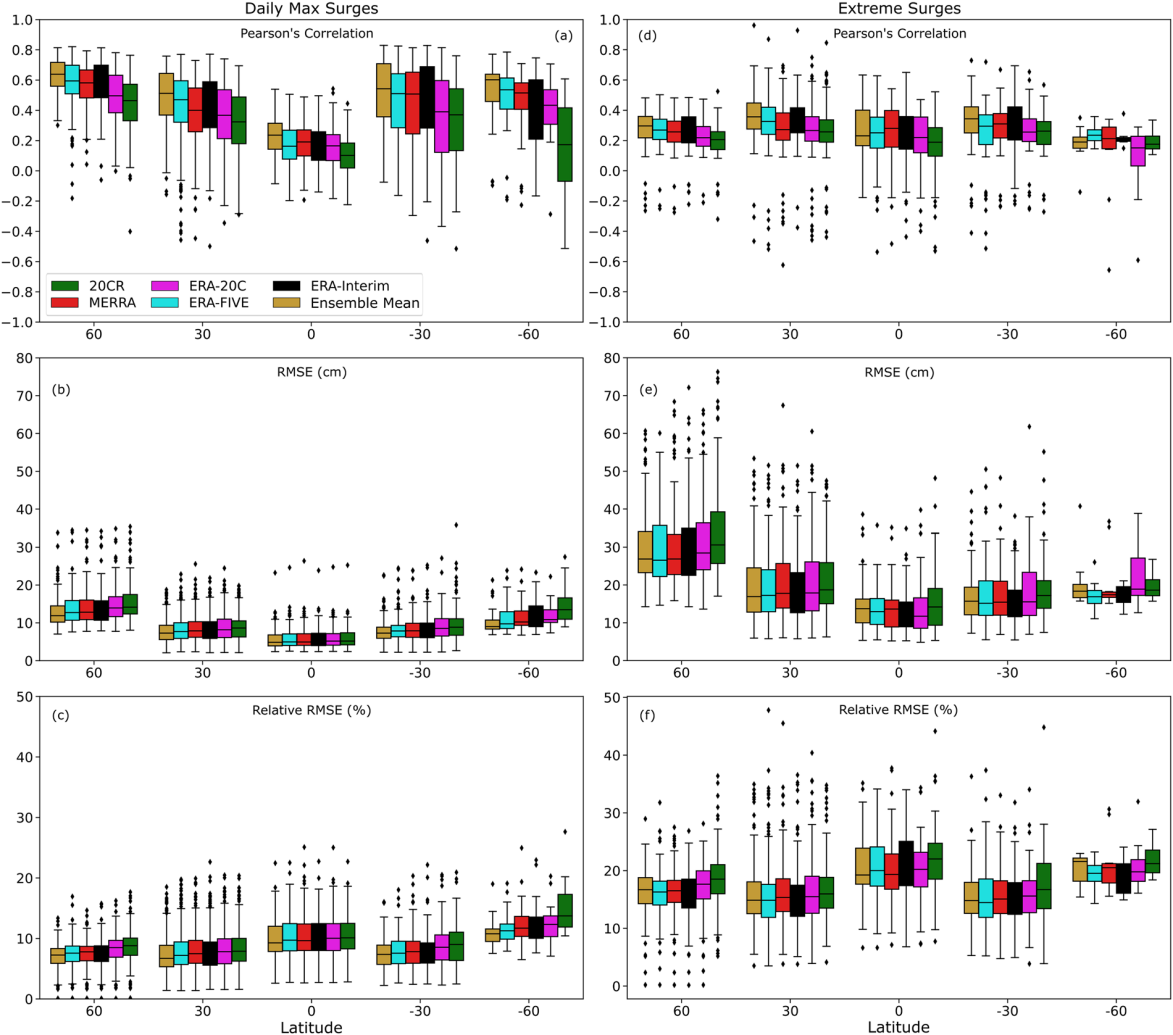
Spatial variation of model performance for different reanalysis products. Validation results for five surge reconstructions and the ensemble mean when using all daily values (**a**–**c**) and for extremes above the 95^th^ percentile (**d**–**f**); color coding denotes different reanalysis products as shown in the legends. (**a**) and (**d**) show results for Pearson’s correlation coefficient, (**b**) and (**e**) for RMSE, and (**c**) and (**f**) for RRMSE.

This is further elaborated in Fig. 5 where we show a spatial distribution (summarized in 30-degree bands) of the validation of all five surge reconstructions in addition to the ensemble mean. The ensemble mean is computed by taking the average of the five surge reconstructions at a daily time step. This time series is then validated against the observed surge. The ensemble mean has relatively higher model accuracy in most places especially in higher latitudes. For future work, a weighted mean ensemble approach^21^ (by weighting the five reconstruction time series based on their validation results) could be tested as opposed to the simple mean ensemble approach used here.

In order to test the difference in performance of our surge reconstructions before and after the satellite era, we selected tide gauges that have data from 1949–2009 and used 1949–1978 and 1979–2009 (with 75% completeness) to represent the pre-satellite era and satellite era respectively. We selected these periods to match overlapping periods of 20-CR and ERA-20C. We validated the surge reconstructions from 20-CR and ERA-20C during these periods and find that out of 49 tide gauges, 31 have a lower RMSE during the satellite era (1979–2009) with an average 4.8% (std = 5%) reduction in RMSE. The remaining 18 tide gauges have an average increase in RMSE of 3.4% (std = 3.9%). RMSE results for these tide gauges are shown in Online-only Table 1.

As outlined above, one advantage of the data-driven models is the ability to not just reconstruct storm surges from multiple reanalysis products, but also over long time periods, which allows more robust analysis of trends, variability, and assessing extreme values. If we only compare results from the centennial 20-CR and ERA-20C reconstructions, ERA-20C leads to better results at approximately 70% of the tide gauges. This is likely due to the fact that 20-CR does not correct for biases in surface pressure observations from ships and buoys^22^ as is done in ERA-20C. If we were to use these two reanalysis products only, we would see a maximum of 20% reduction in model accuracy (compared to using the best of all five reanalyses) for 87% of the tide gauges and a maximum of 40% reduction for 98% of the tide gauges. On average the best of these two reconstructions has 0.8 cm (std = 1.12 cm) higher RMSE than the best reconstruction. When focusing only on extreme events larger than the 95^th^ percentile, the values remain similar; a maximum of 20% reduction for 85% of the tide gauges and a maximum of 40% reduction for 98% of the tide gauges. The global mean increase in RMSE is 2 cm (std = 3 cm) compared to the best surge reconstruction for the extremes.

## Usage Notes

The five surge reconstructions based on the five reanalyses are available for public use at the specified DOIs in Table 2. Users can download the full reconstruction data for all tide gauges for the respective reconstruction. For users interested only in one tide gauge (or few tide gauges), we provide an interactive web-map where users can navigate to download the data manually. An option for bulk download of the full reconstruction is also available through the web-map. The web-map can be accessed through http://gssr.info.

This first version of GSSR provides useful data for certain types of analyses, especially in regions/locations where the model performs well but little observational data is available; this could include, for example, the analysis of longer-term trends, assessing variability and links to large-scale climate patterns, or performing extreme value analysis. However, users should be aware of certain shortcomings and use and interpret the results derived with the GSSR data accordingly. For example, previous work has shown that the quality of centennial reanalysis products declines when going back to the early 20^th^ and 19^th^ centuries^17^. The time periods during which the longer-term surge reconstructions are (more) reliable will be assessed in a follow-up study by comparing our reconstructions against historic sea level data using change point detection techniques, among others. Hence, we recommend that users who use the full centennial surge reconstructions interpret results with caution at this point, especially when assessing non-stationarity (as it may stem from changes in the quality of the reanalysis data over time). Furthermore, our validation shows that the model, in line with previously used global storm surge models, performs poorly in the tropical regions where predictor data shows very little variability and other processes which are not included here may be more relevant (e.g., related to wind-wave and swell activity). However, extreme value analysis as part of flood risk assessments or coastal design and adaptation studies is usually performed on the still water levels and not just the surge component. In T20 we show that the performance metrics increase substantially when our surge reconstructions are combined with predicted tide levels and then compared to observed still water levels, including high correlation and low RMSE for locations in the tropics (see Fig. 8 in T20). Bias correction techniques are also widely applied to derive more robust results from extreme value analyses when using model output data, and have not been explored with the GSSR data. We plan to make regular updates to the web-tool by including new data and products directly derived from the surge reconstructions, including more detailed information on how far back centennial reconstructions can be trusted in different regions, as well as results from assessing trends and performing extreme value analysis on still water levels.

## Data Availability

All scripts used for predictor extraction, predictor pre-processing, model training/validation, and surge reconstruction are available for download under the DOI listed in Table 2.

## Online-only Table

**Online-only Table 1:** Validation of 20-CR and ERA-20C reconstructions validated in the pre-satellite era (1949–1978) and satellite era (1979–2009).

Tide Gauge	20-CR RMSE (m)			ERA-20C RMSE (m)		
	Pre-Satellite Era	Satellite Era	Change (%)	Pre-Satellite Era	Satellite Era	Change (%)
Astoria,or_572a_usa	0.111	0.112	−1.1	0.103	0.106	−2.96
Atlantic_city_264a_usa	0.156	0.157	−0.6	0.119	0.119	0.11
Balboa_302a_panama	0.079	0.080	−1.5	0.073	0.075	−2.26
Boston,ma_741a_usa	0.121	0.119	1.4	0.114	0.111	2.24
Brest_france	0.099	0.089	10.3	0.098	0.089	8.90
Ceuta_ceta_spain	0.076	0.073	3.7	0.063	0.061	2.13
Charleston,sc_261a_usa	0.147	0.146	0.5	0.113	0.110	2.55
Charlottetown,pei_01700_canada	0.174	0.173	0.4	0.157	0.157	−0.04
Coruna_coru_spain	0.091	0.088	3.2	0.078	0.078	0.71
Crescent_city,ca_556a_usa	0.092	0.088	3.4	0.088	0.084	4.74
Cristobal_266a_panama	0.038	0.038	0.1	0.035	0.034	1.12
Cuxhaven_germany	0.301	0.311	−3.6	0.266	0.274	−3.10
Eastport,me_740a_usa	0.136	0.105	22.4	0.139	0.109	21.44
Fort_pulaski,ga_752a_usa	0.203	0.199	2.2	0.130	0.127	1.72
Galveston,pier_21_775a_usa	0.132	0.130	1.2	0.114	0.111	2.45
Gedser_837a_denmark	0.195	0.200	−2.2	0.164	0.168	−2.02
Halifax,ns_00490_canada	0.116	0.115	1.2	0.113	0.111	2.14
Hilo,_hawaii_060a_usa	0.044	0.039	11.4	0.044	0.039	10.28
Honolulu_b,hawaii_057b_usa	0.037	0.037	2.2	0.036	0.036	−0.03
Hornbaek_838a_denmark	0.137	0.139	−1.5	0.128	0.131	−2.11
Ketchikan,ak_571a_usa	0.137	0.124	9.9	0.124	0.111	10.47
Key_west,fl_242a_usa	0.056	0.059	−4.5	0.055	0.058	−5.30
Kwajalein_055a_rep._of_marshall_i	0.037	0.035	7.1	0.036	0.035	4.56
La_coruna_830a_spain	0.089	0.086	3.4	0.079	0.078	1.28
La_jolla,ca_554a_usa	0.052	0.053	−2.7	0.045	0.046	−3.01
Los_angeles,ca_567a_usa	0.052	0.055	−5.6	0.046	0.047	−2.64
Neah_bay,wa_558a_usa	0.099	0.091	7.8	0.110	0.107	3.41
Newlyn_p001_uk	0.107	0.105	1.8	0.111	0.112	−0.28
Newport,ri_253a_usa	0.114	0.110	3.5	0.114	0.110	3.34
New_london,ct_744a_usa	0.127	0.127	0.1	0.119	0.118	1.31
New_york,ny_745a_usa	0.149	0.147	1.8	0.131	0.129	1.80
Pensacola,fl_762a_usa	0.115	0.121	−5.2	0.070	0.077	−10.54
Pointatkinson,bc_07795_canada	0.116	0.112	3.7	0.126	0.123	1.94
Portland,me_252a_usa	0.109	0.093	14.3	0.117	0.103	11.40
Princerupert,bc_09354_canada	0.131	0.131	−0.6	0.128	0.128	0.49
Saintjohn,nb_00065_canada	0.153	0.145	5.5	0.148	0.137	7.45
Santander_ieo_spain	0.075	0.088	−17.1	0.067	0.082	−21.75
San_diego_569a_usa	0.044	0.043	1.2	0.040	0.041	−0.41
San_francisco,ca_551a_usa	0.061	0.062	−1.2	0.053	0.052	1.84
Sitka,ak_559a_usa	0.120	0.116	2.9	0.105	0.104	0.41
St._petersburg,_fl_759a_usa	0.126	0.130	−3.4	0.093	0.094	−1.33
Stockholm_826a_sweden	0.121	0.118	*1.9*	0.119	0.116	2.16
Tarifa_tari_spain	0.106	0.092	13.1	0.076	0.059	22.30
Tofino,bc_08615_canada	0.143	0.143	−0.4	0.138	0.137	0.59
Vancouver,bc_07735_canada	0.131	0.126	3.4	0.118	0.117	0.74
Victoria,bc_543a_canada	0.114	0.110	3.6	0.095	0.092	3.45
Vigo_ieo_spain	0.082	0.087	−6.7	0.080	0.086	−7.51
Wellington_071a_new_zealand	0.082	0.085	−3.4	0.075	0.076	−0.28
Wilmington,nc_750a_usa	0.115	0.116	−0.7	0.110	0.110	0.08
